# Respiratory virology dashboards for primary care and public health insight in England

**DOI:** 10.1371/journal.pone.0357287

**Published:** 2026-09-16

**Authors:** Cecilia Rago, Pushpa Kumarapeli, Harshana Liyanage, José M. Ordóñez-Mena, Uy Hoang, Gavin Jamie, Jessica Smylie, Timea Suli, Katja Hoschler, Anika Singanayagam, Beatrix Kele, Praveen SebastianPillai, Maria Zambon, Simon de Lusignan

**Affiliations:** 1 Nuffield Department of Primary Care Health Sciences, University of Oxford, Oxford, United Kingdom; 2 School of Computer Science and Mathematics, Kingston University London, Kingston upon Thames, United Kingdom; 3 Regions Data Science, Data and Surveillance Services Division, UK Health Security Agency, London, United Kingdom; 4 Respiratory Virus Unit (RVU), UK Health Security Agency, Colindale, United Kingdom; 5 Royal College of General Practitioners, London, United Kingdom; Università degli Studi di Parma: Universita degli Studi di Parma, ITALY

## Abstract

**Background:**

Effective community surveillance of respiratory diseases relies on the timely collection and analysis of high quality clinical and virology data. The aim was to develop interactive dashboards for general practice, public health, and researcher groups. This would improve the visualisation, utilisation, and quality of virology data from an English sentinel network, support disease trend monitoring, early outbreak response, and thereby reduce poor health outcomes and their societal impact.

**Methods:**

The study was conducted in the Oxford-Royal College of General Practitioners (RCGP), Research and Surveillance Centre (RSC) sentinel network sponsored by the UK Health Security Agency (UKHSA). A collaborative user-centred design (UCD) approach was used to identify three use cases for general practices, national public health collaborators, and researchers in the internal team. Reference laboratory virology data, and electronic primary care health records were processed, cleaned, linked, and aggregated in a secure Trusted Research Environment (TRE). Dashboards were developed using Microsoft Power BI (Business Intelligence). The effectiveness, usability, and user satisfaction of the dashboards were not formally evaluated.

**Results:**

Interactive dashboards were co-designed to present spatiotemporal trends of circulating respiratory viruses in the community, in near real-time, with weekly updates on a Wednesday until the previous week ending on Sunday. The dashboard allowed primary care users to filter data to their own results, and it presented key metrics such as virus positivity rates, with benchmarked targets for team incentives. The dashboard also supported public health planning, and research and surveillance activities of the internal RSC team.

**Conclusion:**

The user-centred dashboards created a data feedback loop. Future work requires formal evaluation of usage metrics, user impact and satisfaction, data quality, and the quality of virology samples.

## Introduction

### The use of data visualisation techniques in respiratory disease surveillance worldwide

Data visualisation tools are used in routine surveillance to monitor the spatiotemporal trends of respiratory diseases by clinical characteristics [1]. For over 50 years, the Oxford-Royal College of General Practitioners (RCGP) Research and Surveillance Centre (RSC) has monitored influenza-like illness (ILI) and other respiratory conditions in collaboration with the UK Health Security Agency (UKHSA) and previous national public health bodies [2]. The European Centre for Disease Control Prevention (ECDC) also publish weekly comparisons of respiratory surveillance data by country [3]. Since 1952, the World Health Organization (WHO) has also published a range of respiratory virus trends through their Global Influenza Surveillance & Reporting System (GISRS) [4].

### Developing primary care virology data reporting

The visual representation of public health information supports effective and timely evidence-based decision-making [5]. Since 2014, the Research and Surveillance Centre (RSC) has used visualisation techniques to report trends in the incidence of over 37 monitored conditions [6]. The standard method for evaluating the effectiveness of influenza vaccines is the test-negative case control (TNCC) observational cohort study, where VE (vaccine effectiveness) is measured by: (1 – incidence rate in the vaccinated/ incidence rate in the unvaccinated)*100 [7].

A laboratory confirmed diagnosis of influenza is a key data requirement for VE analysis [8]. In 2018, it was reported that 2,881 of 4,251 (68%) laboratory confirmed virology sample data collected by sentinel general practices in the RSC met data quality standards. This was therefore the rationale to improve data quality using an interactive dashboard for audit and feedback (A&F). A dashboard was created using Tableau to support this and it was found to be effective [9]. Details of the performance indicators that were included in this dashboard are provided in the supplementary file (S1 Fig in S1 File).

The COVID-19 pandemic in 2020 increased the need for virology surveillance data that was timely and that had a bigger sample size. A study published in May 2025 summarised the range of operational changes that took place during this period [10]. While a new virology report that was developed using Microsoft Power BI was felt to be an improvement, the data was at an aggregate level without the practice-specific feedback that was previously found to be valuable [11]. Therefore, there was a need to develop the reporting tools to make them more dynamic and user-friendly.

The aim of this study was to retrospectively describe the development and implementation of virology dashboards for different use cases. This descriptive study may also inform other epidemiologists and dashboard developers in the field. A formal evaluation of the impact of the virology dashboards on usability and user satisfaction was not part of this work.

## Methods

### Identifying user requirements

We adopted a user-centred design (UCD) methodology that aims to create technologies that are intuitive, valuable, and easy to use. The UCD method does this by iteratively prioritising users’ needs and perspectives at all stages of development [12]. We also used the technology acceptance model (TAM) to guide dashboard development. This model posits that perceived usefulness and ease-of-use are key determinants of whether a new technology will be adopted by its intended users [13].

Microsoft’s “build-measure-learn” requirements analysis framework was also incorporated into the dashboard development process, to gather user-requirements from internal and external primary care and public health stakeholders, during meetings held between the 25^th^ of November 2020 and the 2^nd^ of September 2024.

### Data collection from a national swabbing programme

Between the 7^th^ of October 2019 (ISO week 41 of 2019), and the 18^th^ of August 2024 (ISO week 33 of 2024), virology swab samples were collected from patients at sentinel general practices in the Research and Surveillance Centre (RSC). Patient samples were tested as part of the national swabbing program at the UK Health Security Agency (UKHSA) Respiratory Virus Unit (RVU) reference laboratory [14].

Pseudonymised data was accessed for this descriptive study on Friday the 23^rd^ of August 2024 and no authors had access to any information that could identify individual participants during and after data collection. All data were cleaned and analysed within the secure Trusted Research Environment (TRE) of the Oxford-RCGP Clinical Informatics Digital Hub (ORCHID).

Pseudonymised primary care data was collected for surveillance and was therefore processed under Health Service Regulation 3 (Control of Patient Information Regulations 2002) which is renewed annually under Regulation 7 by the Caldicott Guardian at the UK Health Security Agency. No further ethical approval was obtained.

### Algorithm to identify acute respiratory infections in the electronic health record

A clinically curated algorithm called the ARI (acute respiratory infection) hierarchy was derived in the dataset using one categorical column to ensure clinical consistency. One primary diagnosis for each positive or negative test result was assigned, by searching the patient’s electronic health record 10 days before and after the sample was taken, or if this date was missing, the date the sample was received at the UKHSA RVU reference laboratory. If multiple clinical ARI codes were present in the patient’s record, the diagnosis was selected in the following order of priority with one being the highest: 1) influenza-like illness (ILI); 2) exacerbations of asthma and other respiratory disease exacerbations; 3) exacerbations of chronic obstructive pulmonary disease; 4) lower respiratory tract infections (including pneumonia, bronchitis, bronchiolitis, and lower respiratory tract infections not otherwise specified); 5) upper respiratory tract infections (including laryngitis, croup, sinusitis, tonsillitis and pharyngitis, otitis media, and upper respiratory tract infections not otherwise specified); 6) other acute respiratory tract infections, including suspected COVID-19 (coronavirus disease), laboratory or clinically confirmed COVID-19, and acute respiratory tract infections not otherwise specified [15].

The output of the derived ARI hierarchy was the categorical column in the optimised data feed for the dashboard, and this column included a ‘missing data’ value which described results where the latest data extract for the general practice from third party data suppliers was before the most recent reporting period, and therefore, the linked clinical data was unavailable. An ‘ARI Not Specified’ value also described results for symptomatic patients swabbed within 10 days of onset where no ARI diagnostic codes were present in their electronic health record within the search window.

### Virology data import and cleaning

#### Importing the virology data into a secure environment.

Virology sample data files were shared via email from the UK Health Security Agency (UKHSA) to the Research and Surveillance Centre weekly on a Tuesday and Thursday. A zip file was transferred to the secure trusted research environment (TRE) to be unzipped and decrypted. It contained sensitive patient-level virology sample data and the pseudonymised NHS number. Another date-stamped Excel file was transferred to the secure TRE and decrypted. It contained patient-level virology sample data with adenovirus results and no patient identifiers.

Table 1 describes the map of column names in the unzipped and decrypted file received from UKHSA to the column names of the data imported into a Microsoft SQL database in the secure trusted research environment (TRE). Table 1 also includes a description of the data types of the columns stored in the database for the imported data. All files received were imported and stored in tables with the same date stamp as the file. When new virology data files were received from UKHSA, new tables were created to store the data.

**Table 1:** Virology data column mappings from the unzipped decrypted file to the imported table in the secure TRE.

UKHSA column name	TRE column name	TRE column data type
NHSnumber *(pseudonymised)*	NHSNumber *(pseudonymised)*	*Varchar*(128), not null
LID	LID	*Varchar*(2), not null
LPERIOD	LPERIOD	*Smallint*, not null
ORDNB	ORDNB	*Smallint*, not null
SPSEQ	SPSEQ	*Smallint*, not null
SPNB	SPNB	*Varchar*(4), not null
WEEK_NO_OF_RECEIPT	WEEK_NO_OF_RECEIPT	*Smallint*, not null
PROJNB	PROJNB	*Varchar*(8), not null
RVU_NO	RVU_NO	*Varchar*(12), not null
DATE_RECEIVED	DATE_RECEIVED	*Date*, not null
SEX	SEX	*Varchar*(1), not null
Age	Age	*Numeric*(5,2), not null
SAMPLE_DATE	SAMPLE_DATE	*Date*, null
TYPE_OF_SAMPLE	TYPE_OF_SAMPLE	*Varchar*(50), not null
FLREAL	FLREAL	*Varchar*(50), not null
FLRSRL	FLRSRL	*Varchar*(255),not null
ECOM	ECOM	*Varchar*(257), not null
FLCOM	FLCOM	*Varchar*(256), not null
FLCORC	FLCORC	*Varchar*(1000), not null
FLCORC_STATUS	FLCORC_STATUS	*Varchar*(10), not null
SeasonalCoronavirusResult	SeasonalCoronavirusResult	*Varchar*(255), not null
FLU_COMMENT	FLU_COMMENT	*Varchar*(255), not null
ADENO_HEXON_QS	ADENO_HEXON_QS	*Varchar*(255), not null
ADENO_FLCOM5_COMMENT	ADENO_FLCOM5_COMMENT	*Varchar*(1000), not null
ORIGIN	ORIGIN	*Varchar*(6), not null
ID	ID	*Varchar*(30), not null
SURGERY	SURGERY	*Varchar*(40), not null
ADDRESS1	ADDRESS1	*Varchar*(40), not null
ADDRESS2	ADDRESS2	*Varchar*(40), not null
ADDRESS3	ADDRESS3	*Varchar*(40), not null
ZIP	ZIP	*Varchar*(8), not null
CITY	CITY	*Varchar*(40), not null
REGION	REGION	*Varchar*(15), not null
TEL	TEL	*Varchar*(50), not null
Order	Order	*Smallint*, null
ONSET	ONSET	*Varchar*(20), not null
CMO_RISKGRP	CMO_RISKGRP	*Varchar*(50), not null
TASTE_SMELL	TASTE_SMELL	*Varchar*(50), not null
DOB	DOB	*Varchar*(40), not null
FULLDB	FULLDB	*Varchar*(40), not null
Current_Seasonal_Vaccine	Current_Seasonal_Vaccine	*Varchar*(30), not null
Current_Seasonal_Vaccine_date	Current_Seasonal_Vaccine_Date	*Varchar*(20), not null
Current_VACCINE_ADMINISTRATION	Current_VACCINE_ADMINISTRATION	*Varchar*(25), not null
Previous Flu Vacc	Previous Flu Vacc	*Varchar*(25), not null
Previous vac Date1	Previous_Flu_Vacc_Date1	*Varchar*(50), not null
Previous Flu Vac Admin	Previous_Flu_Vacc_Admin	*Varchar*(25), not null
REFERRING_LAB_ID	REFERRING_LAB_ID	*Varchar*(30), not null
Lab Comment	Lab Comment	*Varchar*(255), not null
PendingId	PendingId	*Smallint*, not null
PracticeID	PracticeID	*Varchar*(10), not null
PrimaryID	PrimaryID	*Varchar*(50), not null
SecondaryID	SecondaryID	*Varchar*(50), not null
Gender	Gender	*Varchar*(1), not null
SavLivesNHSNumr *(pseudonymised)*	SavLivesNHSNumr *(pseudonymised)*	*Varchar*(128), not null
GpPostcode	GpPostcode	*Varchar*(8), not null
VoucherCode	VoucherCode	*Varchar*(50), not null
DateOfIllnessOnset	DateOfIllnessOnset	*Varchar*(50), not null
Ethnicity	Ethnicity	*Varchar*(5), not null
RecentSymptoms	RecentSymptoms	*Varchar*(255), not null
InHospital	InHospital	*Varchar*(3), not null
FitAndWell	FitAndWell	*Varchar*(3), not null
FluRisk	FluRisk	*Varchar*(3), not null
HealthCareWorker	HealthCareWorker	*Varchar*(3), not null
CareHomeWorker	CareHomeWorker	*Varchar*(3), not null
Household	Household	*Varchar*(100), not null
Pregnant	Pregnant	*Varchar*(3), not null
OtherNumber	OtherNumber	*Varchar*(50), not null
FluVaccinated	FluVaccinated	*Varchar*(50), not null
FluVaccinatedDate	FluVaccinatedDate	*Varchar*(50), not null
FluWhereVaccinated	FluWhereVaccinated	*Varchar*(50), not null
FluVaccinatedOther	FluVaccinatedOther	*Varchar*(255), not null
HwInHousehold	HwInHousehold	*Varchar*(50), not null
ChwInHousehold	ChwInHousehold	*Varchar*(50), not null
RecentSymptomsOther	RecentSymptomsOther	*Varchar*(255), not null
HouseholdNew	HouseholdNew	*Varchar*(50), not null
HouseholdNewOther	HouseholdNewOther	*Varchar*(255), not null
HouseholdNumbers	HouseholdNumbers	*Varchar*(50), not null
ContactWithOthers	ContactWithOthers	*Varchar*(50), not null
ContactWithOthersLoc	ContactWithOthersLoc	*Varchar*(50), not null
ContactLocOther	ContactLocOther	*Varchar*(50), not null
RecentHospitalVisit	RecentHospitalVisit	*Varchar*(50), not null
PublicTransport	PublicTransport	*Varchar*(50), not null
PublicTransportTime	PublicTransportTime	*Varchar*(50), not null
TravelAbroad	TravelAbroad	*Varchar*(50), not null
TravelAbroadCountries	TravelAbroadCountries	*Varchar*(50), not null
TravelAbroadTransport	TravelAbroadTransport	*Varchar*(50), not null
Excursions	Excursions	*Varchar*(50), not null
Visit	Visit	*Varchar*(50), not null
AttendingWorkOrCollege	AttendingWorkOrCollege	*Varchar*(50), not null
WorkContact	WorkContact	*Varchar*(50), not null
WorkChildren	WorkChildren	*Varchar*(50), not null
WorkLocation	WorkLocation	*Varchar*(50), not null
WorkType	WorkType	*Varchar*(50), not null
WorkTypeVehicle	WorkTypeVehicle	*Varchar*(50), not null
WorkTypeOther	WorkTypeOther	*Varchar*(50), not null
ProfessionalContact	ProfessionalContact	*Varchar*(50), not null
[N/a: not available]	OnsetDate	*Computed*, date, null
[N/a: not available]	CurrentSeasonalVaccineDate	*Computed*, date, null
[N/a: not available]	PreviousFluVaccineDate	*Computed*, date, null
[N/a: not available]	OnsetDate2	*Computed*, date, null

An explanation of the map of the columns from the raw data received from the UK Health Security Agency (UKHSA) to the columns and data types of the data stored in the trusted research environment (TRE).

Sometimes, the decrypted file contained a slightly different range of columns. The table structure that was created to receive each new dataset was adapted according to the columns received. Only distinct rows were kept in the table when the data was imported and this step removed duplicates. The format of columns that held dates was cleaned by converting the format to the ‘date’ data type and blank spaces were trimmed from the columns that contain them.

After the 6th of January 2022, historical data was not contained in the data files received from the UKHSA. We therefore used the data received on the 6th of January 2022 and joined it to the latest extract prior to this date to combine it with the historical data.

#### Data cleaning of virology laboratory free-text to binary indicators.

Table 2 describes the virology data used for linkage and data aggregation for the virology dashboards. The cleaned data was stored in a view in the Microsoft SQL server database. A mapping table was created in the secure trusted research environment (TRE) of virus free-text values to binary indicators for positive or negative results (1 = positive, 0 = negative). Any new free-text strings were added to the mapping table. The following binary indicator columns were included in the cleaned view of the data: NotTested; NoVirusDetected; InfluenzaA; InfluenzaASubtypeH3; InfluenzaASubtypeH1InfluenxaASubtypeOtherorUnknown; InfluenzaB; LAIV; hMPV; RSV; RSVSubtypeA; RSVSubtypeB; RSVSubtypeOtherOrUnknown; COVID19; OtherCoronavirus; Adenovirus; HumanRhinovirus; Enterovirus; NotTestedReason.

**Table 2:** Virology data column mappings from the imported data to the cleaned table in the secure TRE.

UKHSA column name	TRE column name
RVU_NO	PHEID
ORIGIN	SamplePracticeID
NHSnumber *(pseudonymised)*	NHSNumber *(pseudonymised)*
Age	Age
Sex	Sex
SAMPLE_DATE	DateOfSampleTaken
DATE_RECEIVED	DateOfSampleReceived
OnsetDate	DateOfOnset
[N/a: not available]	NotTested
[N/a: not available]	NoVirusDetected
[N/a: not available]	InfluenzaA
[N/a: not available]	InfluenzaASubtypeH3
[N/a: not available]	InfluenzaASubtypeH1
[N/a: not available]	InfluenzaASubtypeOtherOrUnknown
[N/a: not available]	InfluenzaB
[N/a: not available]	LAIV
[N/a: not available]	hMPV
[N/a: not available]	RSV
[N/a: not available]	RSVSubtypeA
[N/a: not available]	RSVSubtypeB
[N/a: not available]	RSVSubtypeOtherOrUnknown
[N/a: not available]	COVID19
[N/a: not available]	OtherCoronavirus
[N/a: not available]	Adenovirus
[N/a: not available]	HumanRhinovirus
[N/a: not available]	Enterovirus
[N/a: not available]	Result
[N/a: not available]	NotTestedReason
PROJNB	SampleSource

An explanation of the column mappings from the raw data received from the UK Health Security Agency (UKHSA) to the cleaned table with the binary results that were derived using a mapping table in the trusted research environment (TRE).

Table S2 in S1 File describes the map of laboratory free-text results to binary indicators that were positive or negative. Table S2 in S1 File shows which file and source column each text value originated from. A cleaned mapping table of practice lab codes to the practice identifier was also maintained in the SQL server database in the trusted research environment (TRE). When the lab code in the virology data received did not match the known lab code known for the practice, the lab code for the practice that already existed in the virology data record was prioritised.

### Adapting the data transformation pipeline to meet user needs

The virology dashboards were designed in response to specific data availability challenges, and user feedback. To calculate swabbing rates that were included in the previous virology dashboard and report, the registered practice population data (the denominator) was required. However, if the practice denominator data was not extracted from third-party suppliers for the reporting period, practice swabbing rates would be missing. Following practice feedback about this limitation which prevented them from viewing their data, the new virology dashboard was designed to present a time-series of swab counts instead of swabbing rates. This ensured that all virology data for participating practices were presented in the visualisations.

### Dashboard development and deployment

**Fig 1:**
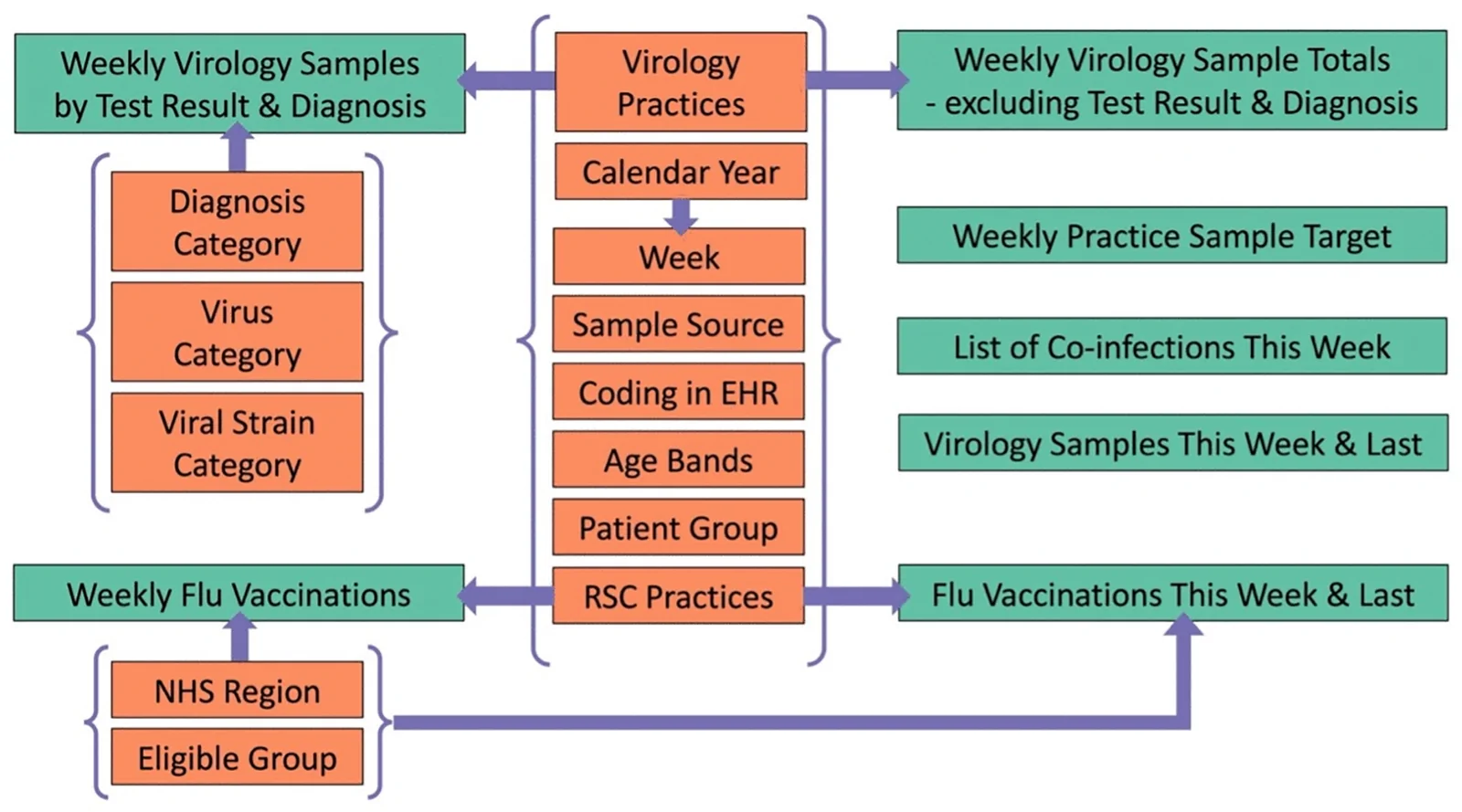
Virology dashboard data model. Schematic diagram of the data model that facilitated user-centric interaction in the virology dashboard.

A central part of the data cleaning and transformation pipeline was the secure linkage of virology data to patient primary care electronic health records using the pseudonymised NHS number. Cleaned binary virology test results, and acute respiratory infection diagnoses were counted and aggregated by week and general practice in the trusted research environment (TRE), which was an optimised format for interactive visualisation. Aggregated tables were exported to an internet-connected SQL server database and were imported into a Microsoft Power BI data model for further transformation, to build and deploy the dashboard. The software choice changed from Tableau to Microsoft Power BI to align with other well-established dashboards from the WHO and for ease of use. A schematic diagram of the data model for the virology dashboard is shown (Fig 1). A unified modelling language (UML) framework described the key entities of the data modelling process [16].

The green-labelled boxes (Fig 1) represent the data tables in the virology dashboard data model. The orange-labelled boxes represent the reference tables. The purple-labelled arrows represent the necessary “one to many” relationship, which is a data modelling term to describe that a record in a reference table relate to multiple records of the same entity in a data table, such as calendar year and week. These were engineered between reference and summary count data tables to facilitate the synchronisation of the end-user’s selections across the visual dashboard display. The reference tables within purple braces describe a group of reference tables with the same relationship with the data table.

A formal evaluation of the dashboard’s effectiveness, user impact, usability or user satisfaction was not conducted.

## Results

There is no data reported on the impact of the virology dashboards on user satisfaction, usability, and effectiveness, such as the number clicks or the number of users. Table S1 in the S1 File describes the results of user requirements analysis.

### Near real-time dashboard updates

In 2022, the virology dashboards that were co-designed were published using Microsoft Power BI for sentinel general practices, the UK Health Security Agency (UKHSA), and Research and Surveillance Centre (RSC) teams. The patient population increased from 2,820,097 patients in ISO week 41, 2019–17,211,993 in ISO week 33, 2024 [6]. The circulating respiratory viruses that were presented by the dashboards was previously reported (10). In 2023, influenza vaccine coverage by clinical risk group was an additional feature of the dashboards [17]. The links to view the dashboard for sentinel general practices, UKHSA and RSC research team use cases are provided in page seven of the supplementary file.

### General practice use case for local primary care insight

The visuals designed for general practice stakeholders are described in this section. The interactive practice-specific view of the data was a key feature, while unfiltered versions of the data supported the UKHSA and RSC use cases.

#### Swab frequency.

**Fig 2:**
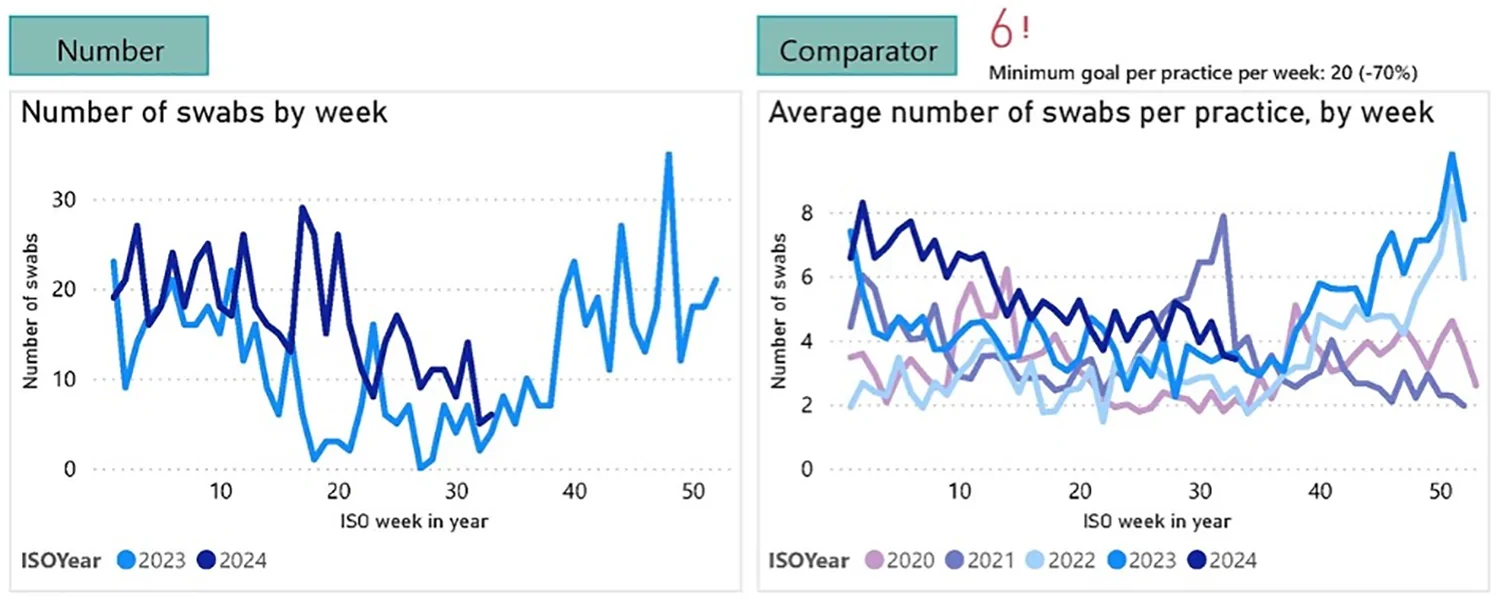
Swabs received at the UKHSA Reference Virology Unit (RVU) reference laboratory. The weekly time-series of swabs received were filtered for a model general practice (left), compared to the average swab count per practice by year (right). The data presented was for two calendar years, and the practice collected on average more swabs than participating practices in the RSC network.

The number of the swabs that were received at the UK Health Security Agency (UKHSA) Respiratory Virus Unit (RVU) reference lab is shown (Fig 2). A line graph compared swab counts for the current year (dark blue) to the previous year (lighter blue). An adjacent graphic to the right showed a comparison of swab counts at the practice selected to the weekly average number per practice for all participating practices. A sequential colour scheme was intended to visualise the yearly chronology. The 2024 calendar year had on average the highest swab frequency. The indicator above the graphic on the right compared the practice frequencies to a pre-specified target where red (with an exclamation sign) indicated that the swab count was below the target, and green (with a tick sign) indicated that the swab count was at or above the target.

#### Circulating viral pathogens.

**Fig 3:**
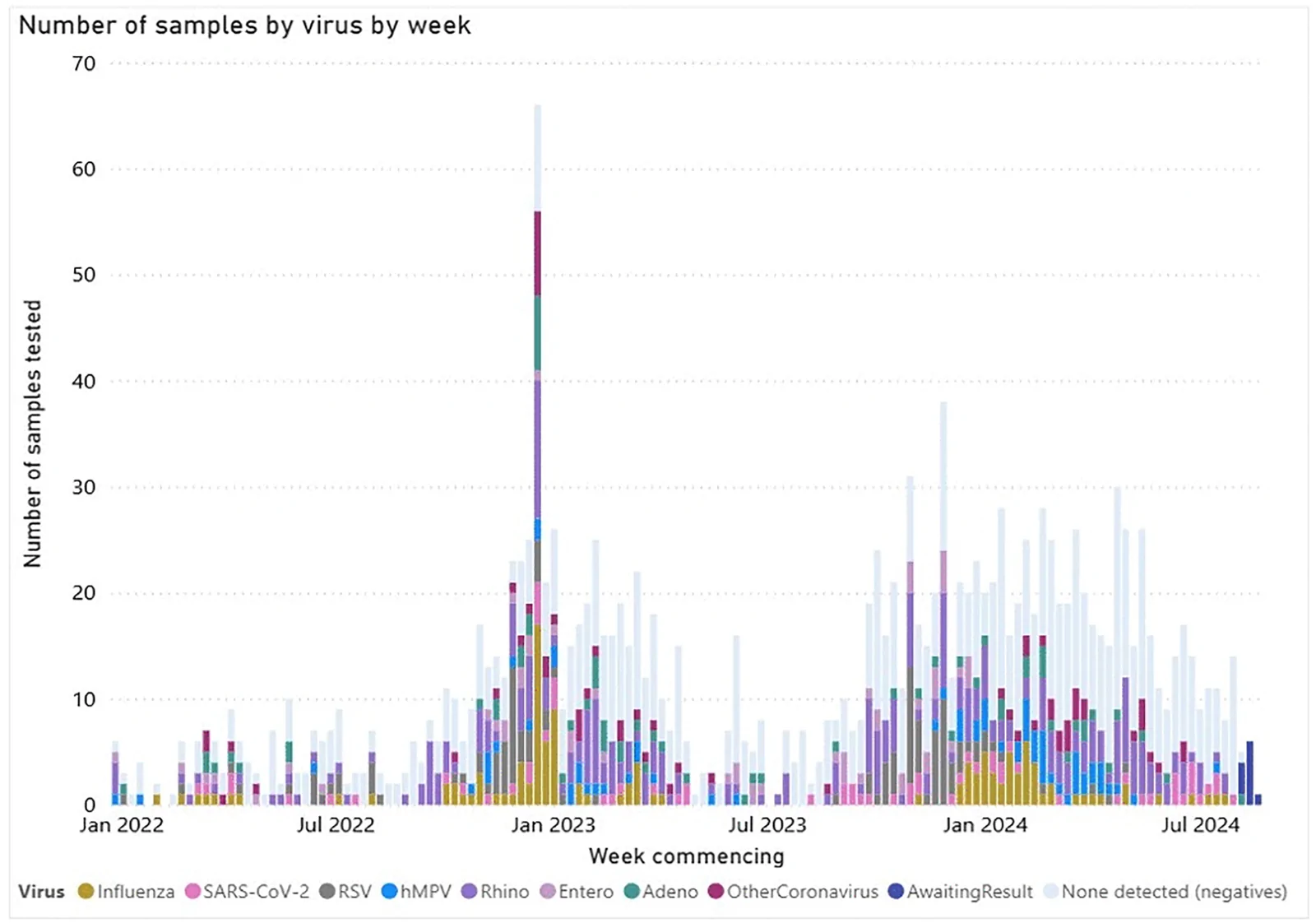
Virology sample test results by virus by week. This graph is a stacked bar chart of virology sample test results by virus by week. It shows an improved legend, and a de-emphasis of samples that tested negative (in faint blue) for a model practice.

The count of each virus detected was displayed in stacked bar charts (Fig 3), and these were: SARS-CoV-2; influenza; RSV; hMPV; seasonal coronaviruses; adenovirus; rhinovirus; enterovirus; negatives; and samples awaiting results. From 2023, negative results were de-emphasized using a faint-blue bar.

### UK Health Security Agency (UKHSA) use case for public health planning

The visuals designed for UK Health Security Agency stakeholders are described in this section. Users could filter the visuals for a specific range, region, or clinical characteristic that they selected.

#### Virus positivity.

**Fig 4:**
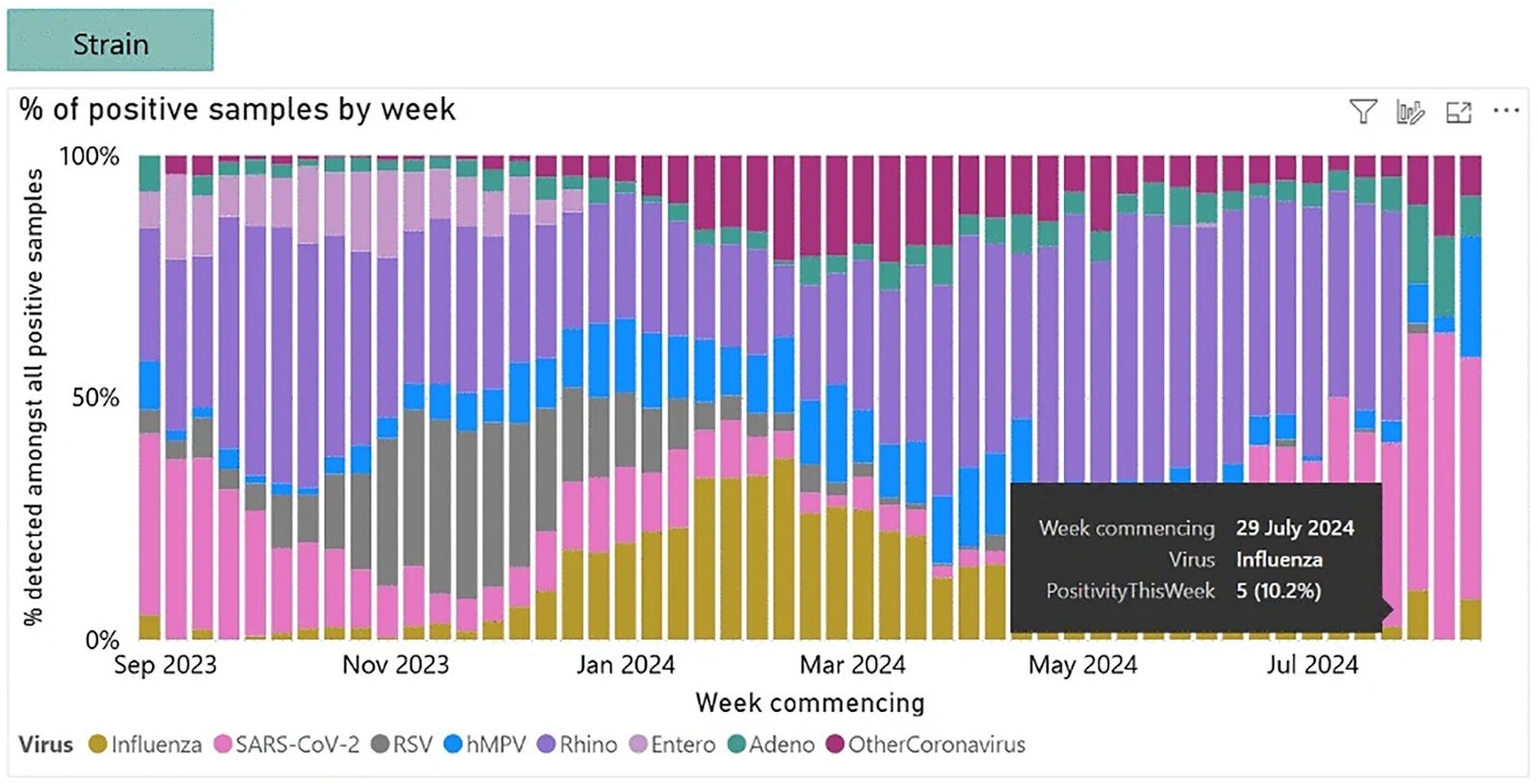
The percentage of positive test results by virus. A green-labelled button above the top left of the 100% stacked bar chart of the percentage of positive test results by virus, allowed the user to toggle the view to see a more granular visualisation by virus strain.

**Fig 5:**
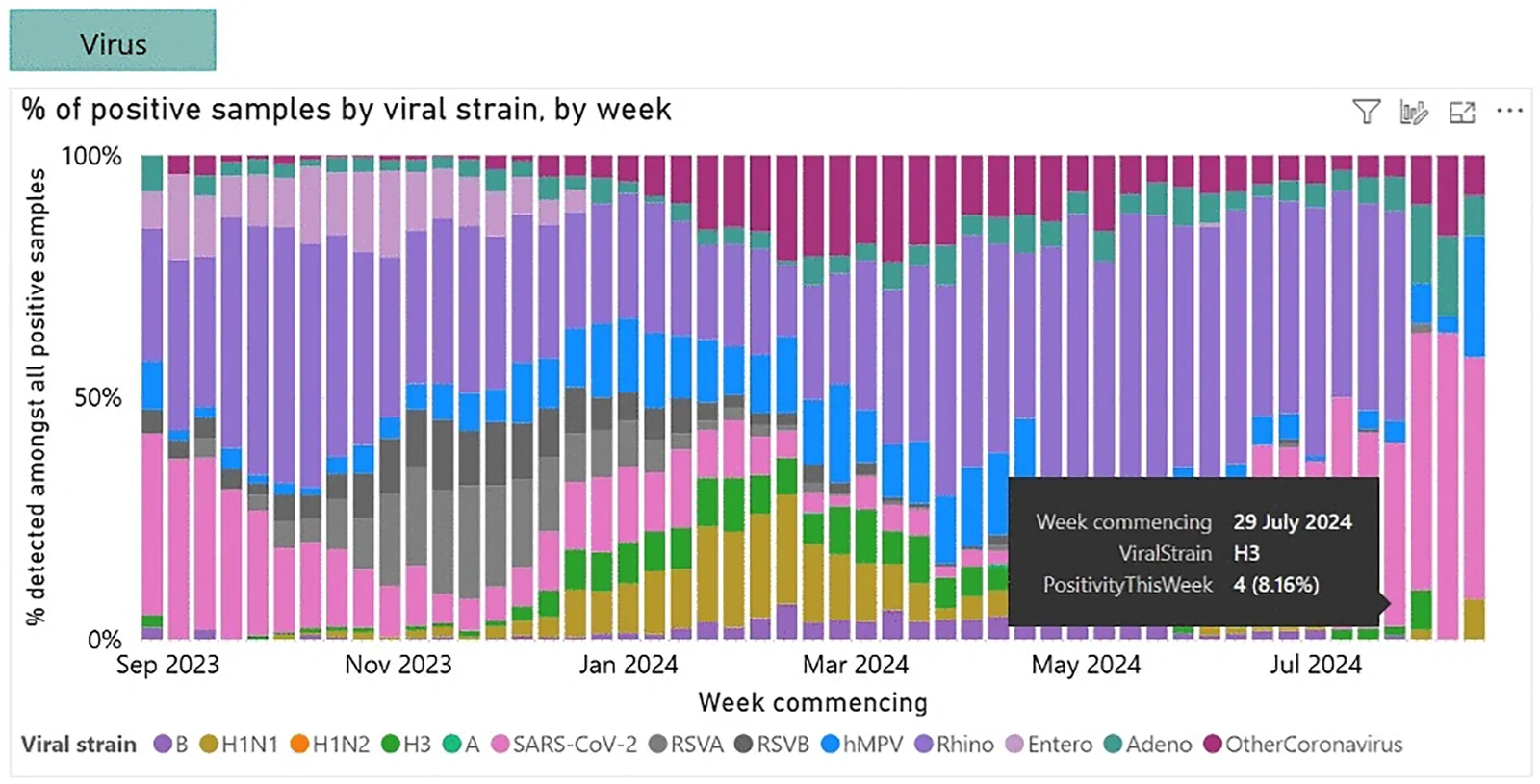
The percentage of positive test results by virus strain. A green-labelled button above the top left of the 100% stacked bar chart of the percentage of positive test results by virus strain, allowed the user to toggle the view to see a less granular visualisation by virus.

The percentage of positive test results by virus was presented on 100% stacked bar charts (Fig 4). The visual could be toggled by clicking a green-labelled button to view the percentage of positive samples by viral strain (Fig 5). The legend shows positive results for: B (influenza B); H1N1 (influenza A (H1N1)pdm09); H3 (influenza A (H3N2)), A (influenza A (unknown subtype)), RSVA (respiratory syncytial virus A); and RSVB (respiratory syncytial virus B). From the 2^nd^ of October 2023 to the 25^th^ of February 2024, SARS-CoV-2 peaked, followed by RSV, and influenza.

#### Co-infections.

A list of patient swab sample results, where more than one positive virus was detected, was co-designed to be displayed in the dashboard to explain the instances where the sum of positives and negatives exceeded the total count of swab samples.

#### Virus detection by age group.

**Fig 6:**
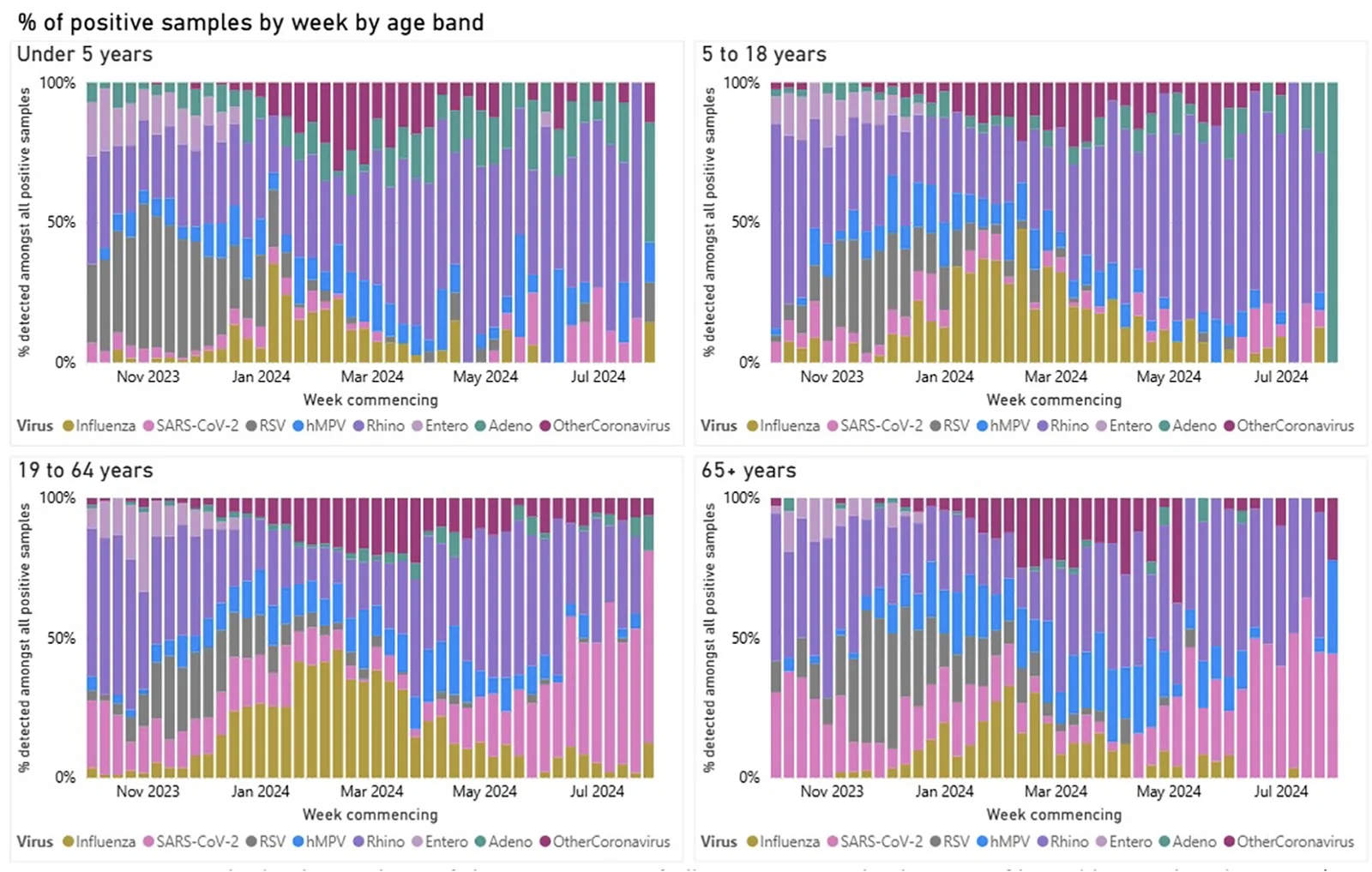
The percentage of positive samples by virus filtered by age-band. 100% stacked bar charts of the percentage of positive samples by virus for the age-bands that the user selected: < 5 yrs (top left), 5-18 yrs (top right), 19-64 yrs (bottom left), 65 + yrs (bottom right).

The proportion of viruses detected by age band was visualised by the user’s selection (Fig 6). Between the 2^nd^ of October 2023 and the 4^th^ of August 2024, a higher proportion of influenza viruses were detected in children aged 5–18, and adults aged 19–64 years, compared to other age bands. RSV was greatest in infants under 5 and adults aged 65 + , while SARS-CoV-2 was detected mostly in people aged 19–64 and 65 years and over.

### RSC team use case for virology sample surveillance and research

This section describes the visualisations designed for the RSC surveillance team. They could monitor sample volumes by geographical region and sample source with the option for data downloads and exploratory analysis for RSC researchers.

In the supplementary file, (Fig S2 in S1 File) shows an unfiltered line graph (of Fig 2) that presents the weekly frequency of swabs collected by all participating practices in the virology swabbing programme. The stacked bar graph of the virus detections by week was also presented (Fig S3in S1 File), and the red box highlights the stepwise increase in the volume of samples collected from October 2023 to August 2024. The percentage of samples that tested positive for any of the viruses tested was also presented (Fig S4 in S1 File).

## Discussion

### Strengths and limitations

The main strength of the virology dashboard development process was the real-world implementation, data cleaning and transformation logic, and co-design approach. A zoonotic swine influenza A infection was detected in a virology sample from a sentinel general practice in 2023, and this prompted the testing of close contacts and an expansion of sentinel swabbing in the surrounding area [18].

The main limitations were that the public accessibility of the Microsoft Power BI dashboards was compromised by website downtime. In addition, usage metrics were not collected, therefore, the usability, user satisfaction and impact of the virology dashboards could not be formally evaluated. Furthermore, sample volumes increased in the 2022/23 and 2023/24 seasons compared to the 2021/22 season; but we do not know whether the development of the virology dashboards was associated with this increase. There were many other operational changes to develop surveillance capabilities during the study period.

Finally, there is a need to develop data quality indicators to provide practice feedback on virology samples that: a) failed eligibility criteria; b) had missing data for surveillance; and c) failed laboratory quality criteria.

### Comparison with existing literature

Existing literature describes data visualisations as valuable tools to benchmark practice-level data, provide feedback, and aid quality improvement [19]. A previous dashboard implementation study also described that without clear ease-of-use, user engagement may dwindle [20]. Our user-centred dashboard design approach aligns with recommendations in relevant literature [21], [22].

The adaptation to user preferences that was described in this study was similar to another study that responded to user demands by designing their dashboard to report multiple pathogens iteratively, in response to the COVID-19 pandemic [23]. A separate study developed a dashboard to support public health planning following the COVID-19 pandemic, and they reported limited formal testing of dashboard effectiveness [24].

The technology acceptance model (TAM) was previously used by authors of a study of facilitators and barriers to dashboard implementation [25]. Other authors also reported iterative dashboard design approaches, with weekly updates, and a subsequent release of COVID-19 vaccination visualisations when the data was available [26].

## Conclusions

This study described the development and implementation of user-centred virology dashboards that presented data and insight that was publicly available, findable, accessible, and open [27]. Our descriptive study also described replicable methods. This work achieved a key objective to co-design the development of virology dashboards for distinct surveillance and research use cases.

For general practices, tools like these have the potential to transform the role of sentinel surveillance from passive data contribution to active engagement. The future of digital surveillance in primary care depends on active learning health systems, where data provision is met with tangible, useful feedback. While we have internal consensus on the usefulness of the virology dashboards, future research should formally evaluate the impact of the virology dashboard in terms of usability, interoperability, and user satisfaction.

## Supporting information

S1 File Virology dashboard manuscript_supplementary file 20260715.docx. Contains sections with additional information on: baseline virology dashboard used for development; identification of use cases for the virology dashboards; new visualisations; website links for the virology dashboard use cases; and virology data rules for mapping free-text data to binary indicators in the Oxford-RCGP Research and Surveillance Centre (RSC).
(DOCX)

## Abbreviations

Adeno: Adenovirus
ARI: Acute respiratory illness
EHR: Electronic health record
Entero: Enterovirus
hMPV: Human metapneumovirus
ILI: Influenza-like illness
ISO: International organization for standardization
NHS: National Health Service
ORCHID: Oxford-RSC clinical informatics digital hub
RCGP: Royal College of General Practitioners
Rhino: Rhinovirus
RSC: Research and Surveillance Centre
RSV: Respiratory syncytial virus
SARS-CoV-2: Severe acute respiratory syndrome coronavirus 2
TAM: Technology acceptance model
TRE: Trusted research environment
UKHSA: UK Health Security Agency
UCD: User-centred design
UML: Unified modelling language
UX: User experience
