# Supplementary material for: Respiratory virology dashboards for primary care and public health insight in England
Source: PLoS One. 2026 Sep 16;21(9):e0357287. doi: 10.1371/journal.pone.0357287 (PMC13580944; doi:10.1371/journal.pone.0357287)
Supplement: Supplementary file 1 [file pone.0357287.s001.docx]

**Supplementary File**

# Baseline virology dashboard used for development

Fig S1 displays example visuals from the virology dashboard that was created in 2018 for the national sentinel virology surveillance swabbing programme in England. The dashboard used Tableau data visualisation software to present sampling performance indicators to improve the quality of virology data used in vaccine effectiveness analysis. This dashboard included bar graphs for individual practices, alongside comparative bars for the RSC network. Practice staff used a randomly generated six-character alphanumeric key to view their practice-specific dashboard, rather than the publicly available NHS practice ODS code, for information governance purposes.

**Fig S1. The previous virology dashboard presenting influenza vaccinations, virology samples, and data quality indicators**

A: influenza vaccination visuals; B: virology data for eligible and swabbed patients, by clinical characteristics; and C: data quality measures, with samples counted according to swabbing performance indicators.

The dashboard also presented:

- The registered practice population count at a given date (the denominator) in comparison to the denominator reported by NHS England.
- Rates of monitored conditions, including influenza-like illness (ILI) and lower respiratory tract infections (LRTI).
- Episode type categorised according to whether the episode was new (incident), or ongoing, such as a follow-up appointment for an existing infection.
- Weekly year-to-date influenza vaccination coverage rates and counts by age group, and Joint Committee on Vaccination and Immunisation (JCVI) risk group category [1]**.**
- Data quality measures for the recording of events, prescriptions, and influenza-vaccine batch numbers.
- Counts of virology samples taken and received.
- Adverse event rates following influenza vaccination.

Reference:

1.	UK Health Security Agency. Immunisation of individuals with underlying medical conditions: the green book, chapter 7. In: The Green Book [Internet]. UK Health Security Agency; 2013 [cited 2025 Oct 10]. p. 1–8. Available from: https://www.gov.uk/government/publications/immunisation-of-individuals-with-underlying-medical-conditions-the-green-book-chapter-7

# Identification of use cases for the virology dashboards

Table S1 describes the meetings that were held with internal and external stakeholders to conduct requirements analysis for the new virology dashboards. The purpose of the meetings over the years are described in the “Meeting categories” column. Internal stakeholders were practice liaison staff, research fellows, doctoral students, and senior managers. External stakeholders were senior public health epidemiologists. These meetings supported the user-centred co-design of the dashboards to meet the needs of internal and external stakeholders and support the dashboards practical use. One hundred meetings were conducted totalling 55.05 hours; 59% were held in 2023, and 34% of the total meeting time over the years was focused on developing insight and understanding of the data.

**Table S1. The number and duration in hours of meetings held to collect virology dashboard requirements**

| Study period | 2020 | | 2021 | | 2022 | | 2023 | | 2024 | | Total | |
| --- | --- | --- | --- | --- | --- | --- | --- | --- | --- | --- | --- | --- |
| Meeting categories: | n (%) | Hours (%) | n (%) | Hours (%) | n (%) | Hours (%) | n (%) | Hours (%) | n (%) | Hours (%) | Total n (%) | Total Hours (%) |
| Data quality improvements | - | - | - | - | 2 (7) | 2 (11.9) | 2 (3.4) | 0.75 (3) | - | - | 4 (4) | 2.75 (5) |
| Motivating general practice performance | - | - | 2 (50) | 1 (100) | 5 (18.5) | 2.5 (14.9) | 12 (20.3) | 5.75 (19) | - | - | 19 (19) | 9.25 (16.8) |
| Reviewing methods to report virology data | - | - | - | - | 8 (30) | 6.5 (38.6) | 10 (17) | 5.75 (19) | 3 (33.4) | 2 (36.4) | 21 (21) | 14.25 (25.9) |
| Strategic decision-making | - | - | - | - | 7 (26) | 3.3 (19.8) | 13 (22) | 6.5 (22) | 2 (22.2) | 1 (18.2) | 22 (22) | 10.8 (19.6) |
| Insight and understanding of the data | 1 (100) | 1 (100%) | 2 (50) | 1 (100) | 5 (18.5) | 2.5 (14.9) | 22 (37.3) | 11 (37) | 4 (44.4) | 2.5 (45.4) | 34 (34) | 18 (32.7) |
| Total (n) | 1 (100) | 1 (100) | 4 (100) | 2 (100) | 27 (100) | 16.8 (100) | 59 (100) | 29.75 (100) | 9 (100) | 5.5 (100) | 100 (100) | 55.05 (100) |

A description of the key themes arising from the requirements analysis**.**

As part of the requirements analysis, three distinct dashboard use cases were identified:

1. Sentinel General Practices required confidential, practice-level feedback on swabbing activity and positive detections, benchmarked against anonymised network averages, to support quality improvement.
2. The UK Health Security Agency needed a timely, high-level, interactive overview of spatiotemporal trends for national surveillance and public health planning.
3. The Research and Surveillance Centre’s internal team required data visualisations to monitor the near real-time volume and representativeness of collected virology samples to support surveillance and research studies.

New visualisations

**Fig S2. Swabs received at the UKHSA Respiratory Virus Unit (RVU) reference laboratory**

The weekly time series shows swabs received at the UKHSA Reference Virology Unit laboratory, with data unfiltered for all general practices that participated in the virology swabbing programme (left). The average number of swabs per practice is shown (right).

**Fig S3. Virology sample test results by virus by week**

The stacked column chart shows virology sample test results by virus and week, for all practices that participated in the virology swabbing programme. The red box highlights the 2023-24 season, during which the number of virology samples increased compared to previous seasons.

**Fig S4. The percentage of samples testing positive for any virus tested by week**

The stacked column chart shows, by week, the percentage of samples that tested positive for any of the panel of viruses tested, or no virus was detected. The “unknown” category were samples that were awaiting test results.

# Website links for the virology dashboard use cases

- Sentinel general practices:

<https://app.powerbi.com/view?r=eyJrIjoiNzAyNGQxMTYtMjUxMy00NGRlLTgyNTEtMDBmOTgwZDk1NjExIiwidCI6ImNjOTVkZTFiLTk3ZjUtNGY5My1iNGJhLWZlNjhiODUyY2Y5MSJ9>

- UK Health Security Agency:

<https://app.powerbi.com/view?r=eyJrIjoiMzcwYjQxNDgtNGVmYi00ZDc1LTliMGItMDE5NzhhNWI1YTRkIiwidCI6ImNjOTVkZTFiLTk3ZjUtNGY5My1iNGJhLWZlNjhiODUyY2Y5MSJ9>

- RSC practice liaison team:

<https://app.powerbi.com/view?r=eyJrIjoiZDY5YTM4MmItN2Y3Mi00OGRiLWJlMzktNjAyOGQxNWJhY2JkIiwidCI6ImNjOTVkZTFiLTk3ZjUtNGY5My1iNGJhLWZlNjhiODUyY2Y5MSJ9>

# Virology data cleaning rules for mapping free-text data to binary indicators in the Oxford-RCGP Research and Surveillance Centre (RSC)

Please see Table S2 below for the data cleaning logic for mapping laboratory result free text fields to a binary result field. A positive result coded as 1 and a negative result is coded as 0.

**Table S2.** Mapping of UKHSA virology free-text results to binary result columns in the cleaned data in the Trusted Research Environment (TRE).

| ColumnName | ColumnValue | NotTested | NoVirusDetected | InfluenzaA | InfluenzaASubtypeH3 | InfluenzaASubtypeH1 | InfluenzaASubtypeOtherOrUnknown | InfluenzaB | LAIV | hMPV | RSV | RSVSubtypeA | RSVSubtypeB | RSVSubtypeOtherOrUnknown | COVID19 | OtherCoronavirus | Adenovirus | HumanRhinovirus | Enterovirus |
| --- | --- | --- | --- | --- | --- | --- | --- | --- | --- | --- | --- | --- | --- | --- | --- | --- | --- | --- | --- |
| ADENO_FLCOM5_COMMENT | Adeno virus detected in this sample. | 0 | 0 | 0 | 0 | 0 | 0 | 0 | 0 | 0 | 0 | 0 | 0 | 0 | 0 | 0 | 1 | 0 | 0 |
| ADENO_FLCOM5_COMMENT | Adenovirus deteceted in this sample | 0 | 0 | 0 | 0 | 0 | 0 | 0 | 0 | 0 | 0 | 0 | 0 | 0 | 0 | 0 | 1 | 0 | 0 |
| ADENO_FLCOM5_COMMENT | Adenovirus detected | 0 | 0 | 0 | 0 | 0 | 0 | 0 | 0 | 0 | 0 | 0 | 0 | 0 | 0 | 0 | 1 | 0 | 0 |
| ADENO_FLCOM5_COMMENT | Adenovirus detected in sample | 0 | 0 | 0 | 0 | 0 | 0 | 0 | 0 | 0 | 0 | 0 | 0 | 0 | 0 | 0 | 1 | 0 | 0 |
| ADENO_FLCOM5_COMMENT | Adenovirus detected in this sample | 0 | 0 | 0 | 0 | 0 | 0 | 0 | 0 | 0 | 0 | 0 | 0 | 0 | 0 | 0 | 1 | 0 | 0 |
| ADENO_FLCOM5_COMMENT | Adenovirus detected in this sample. | 0 | 0 | 0 | 0 | 0 | 0 | 0 | 0 | 0 | 0 | 0 | 0 | 0 | 0 | 0 | 1 | 0 | 0 |
| ADENO_FLCOM5_COMMENT | Adenovirus detecyed in this sample | 0 | 0 | 0 | 0 | 0 | 0 | 0 | 0 | 0 | 0 | 0 | 0 | 0 | 0 | 0 | 1 | 0 | 0 |
| ADENO_FLCOM5_COMMENT | Adenovirus is detected in this sample | 0 | 0 | 0 | 0 | 0 | 0 | 0 | 0 | 0 | 0 | 0 | 0 | 0 | 0 | 0 | 1 | 0 | 0 |
| ADENO_FLCOM5_COMMENT | Adenovrius detected in this sample | 0 | 0 | 0 | 0 | 0 | 0 | 0 | 0 | 0 | 0 | 0 | 0 | 0 | 0 | 0 | 1 | 0 | 0 |
| ADENO_FLCOM5_COMMENT | Adnovirus detected in this sample | 0 | 0 | 0 | 0 | 0 | 0 | 0 | 0 | 0 | 0 | 0 | 0 | 0 | 0 | 0 | 1 | 0 | 0 |
| ADENO_FLCOM5_COMMENT | No Adeno irus detected in this sample. | 0 | 1 | 0 | 0 | 0 | 0 | 0 | 0 | 0 | 0 | 0 | 0 | 0 | 0 | 0 | 0 | 0 | 0 |
| ADENO_FLCOM5_COMMENT | No Adeno virus detected in this sample. | 0 | 1 | 0 | 0 | 0 | 0 | 0 | 0 | 0 | 0 | 0 | 0 | 0 | 0 | 0 | 0 | 0 | 0 |
| ADENO_FLCOM5_COMMENT | No Adenorvirus detected in this sample. | 0 | 1 | 0 | 0 | 0 | 0 | 0 | 0 | 0 | 0 | 0 | 0 | 0 | 0 | 0 | 0 | 0 | 0 |
| ADENO_FLCOM5_COMMENT | No Adenovirus detected in this sample. | 0 | 1 | 0 | 0 | 0 | 0 | 0 | 0 | 0 | 0 | 0 | 0 | 0 | 0 | 0 | 0 | 0 | 0 |
| ADENO_FLCOM5_COMMENT | Not tested | 1 | 0 | 0 | 0 | 0 | 0 | 0 | 0 | 0 | 0 | 0 | 0 | 0 | 0 | 0 | 0 | 0 | 0 |
| ADENO_FLCOM5_COMMENT | Sample not tested. The swab was received dry and is therefore unsuitable for testing. | 1 | 0 | 0 | 0 | 0 | 0 | 0 | 0 | 0 | 0 | 0 | 0 | 0 | 0 | 0 | 0 | 0 | 0 |
| ADENO_FLCOM5_COMMENT | Sample not tested. This sample leaked in transit and was therefore unsuitable for testing. | 1 | 0 | 0 | 0 | 0 | 0 | 0 | 0 | 0 | 0 | 0 | 0 | 0 | 0 | 0 | 0 | 0 | 0 |
| ADENO_FLCOM5_COMMENT | Sanger sequencing of a region of the hexon gene failed to generate sufficient sequence to determine adenovirus species or genotype. | 0 | 0 | 0 | 0 | 0 | 0 | 0 | 0 | 0 | 0 | 0 | 0 | 0 | 0 | 0 | 1 | 0 | 0 |
| ADENO_FLCOM5_COMMENT | Sanger sequencing of a region of the the hexon gene indicates the sequence has close similarity to adenovirus genotype 3, species B | 0 | 0 | 0 | 0 | 0 | 0 | 0 | 0 | 0 | 0 | 0 | 0 | 0 | 0 | 0 | 1 | 0 | 0 |
| ADENO_FLCOM5_COMMENT | Sanger sequencing of a region of the the hexon gene indicates the sequence has close similarity to adenovirus genotype 5, species C | 0 | 0 | 0 | 0 | 0 | 0 | 0 | 0 | 0 | 0 | 0 | 0 | 0 | 0 | 0 | 1 | 0 | 0 |
| ADENO_FLCOM5_COMMENT | The onset date is missing for this patient. It is extremely important to record the date of symptom onset on the form. This is to ensure that we can compare estimates of vaccine effectiveness (VE) for influenza vaccines between countries, which requires | 0 | 0 | 0 | 0 | 0 | 0 | 0 | 0 | 0 | 0 | 0 | 0 | 0 | 0 | 0 | 0 | 0 | 0 |
| ADENO_FLCOM5_COMMENT | The onset date is missing for this patient. t is extremely important to record the date of symptom onset on the form. This is to ensure that we can compare estimates of vaccine effectiveness (VE) for influenza vaccines between countries, which requires | 0 | 1 | 0 | 0 | 0 | 0 | 0 | 0 | 0 | 0 | 0 | 0 | 0 | 0 | 0 | 0 | 0 | 0 |
| ADENO_FLCOM5_COMMENT | The sample collection date is missing for this sample. It is extremely important to record the date of sample collection on the form. This is to ensure that we can compare estimates of vaccine effectiveness (VE) for influenza vaccines between countries | 0 | 0 | 0 | 0 | 0 | 0 | 0 | 0 | 0 | 0 | 0 | 0 | 0 | 0 | 0 | 0 | 0 | 0 |
| ADENO_HEXON_QS | #d | 0 | 0 | 0 | 0 | 0 | 0 | 0 | 0 | 0 | 0 | 0 | 0 | 0 | 0 | 0 | 1 | 0 | 0 |
| ADENO_HEXON_QS | #nt | 1 | 0 | 0 | 0 | 0 | 0 | 0 | 0 | 0 | 0 | 0 | 0 | 0 | 0 | 0 | 0 | 0 | 0 |
| ADENO_HEXON_QS | #nvd | 0 | 1 | 0 | 0 | 0 | 0 | 0 | 0 | 0 | 0 | 0 | 0 | 0 | 0 | 0 | 0 | 0 | 0 |
| ECOM | #nt10 | 1 | 0 | 0 | 0 | 0 | 0 | 0 | 0 | 0 | 0 | 0 | 0 | 0 | 0 | 0 | 0 | 0 | 0 |
| ECOM | #nt3 | 1 | 0 | 0 | 0 | 0 | 0 | 0 | 0 | 0 | 0 | 0 | 0 | 0 | 0 | 0 | 0 | 0 | 0 |
| ECOM | #nt4 | 1 | 0 | 0 | 0 | 0 | 0 | 0 | 0 | 0 | 0 | 0 | 0 | 0 | 0 | 0 | 0 | 0 | 0 |
| ECOM | #nt5 | 1 | 0 | 0 | 0 | 0 | 0 | 0 | 0 | 0 | 0 | 0 | 0 | 0 | 0 | 0 | 0 | 0 | 0 |
| ECOM | #nt6 | 1 | 0 | 0 | 0 | 0 | 0 | 0 | 0 | 0 | 0 | 0 | 0 | 0 | 0 | 0 | 0 | 0 | 0 |
| ECOM | #nt7 | 1 | 0 | 0 | 0 | 0 | 0 | 0 | 0 | 0 | 0 | 0 | 0 | 0 | 0 | 0 | 0 | 0 | 0 |
| ECOM | #nt8Specimen archived without testing at your request | 1 | 0 | 0 | 0 | 0 | 0 | 0 | 0 | 0 | 0 | 0 | 0 | 0 | 0 | 0 | 0 | 0 | 0 |
| ECOM | #nt9 | 1 | 0 | 0 | 0 | 0 | 0 | 0 | 0 | 0 | 0 | 0 | 0 | 0 | 0 | 0 | 0 | 0 | 0 |
| ENPCR | #d | 0 | 0 | 0 | 0 | 0 | 0 | 0 | 0 | 0 | 0 | 0 | 0 | 0 | 0 | 0 | 0 | 0 | 1 |
| ENPCR | #inv | 0 | 0 | 0 | 0 | 0 | 0 | 0 | 0 | 0 | 0 | 0 | 0 | 0 | 0 | 0 | 0 | 0 | 0 |
| ENPCR | #n | 0 | 1 | 0 | 0 | 0 | 0 | 0 | 0 | 0 | 0 | 0 | 0 | 0 | 0 | 0 | 0 | 0 | 0 |
| FLCOM | #bv | 0 | 0 | 0 | 0 | 0 | 0 | 1 | 0 | 0 | 0 | 0 | 0 | 0 | 0 | 0 | 0 | 0 | 0 |
| FLCOM | #Bvrsvb | 0 | 0 | 0 | 0 | 0 | 0 | 1 | 0 | 0 | 1 | 0 | 1 | 0 | 0 | 0 | 0 | 0 | 0 |
| FLCOM | #flubv | 0 | 0 | 0 | 0 | 0 | 0 | 1 | 0 | 0 | 0 | 0 | 0 | 0 | 0 | 0 | 0 | 0 | 0 |
| FLCOM | #h1pdm | 0 | 0 | 1 | 0 | 1 | 0 | 0 | 0 | 0 | 0 | 0 | 0 | 0 | 0 | 0 | 0 | 0 | 0 |
| FLCOM | #h1pdmhmpa | 0 | 0 | 1 | 0 | 1 | 0 | 0 | 0 | 1 | 0 | 0 | 0 | 0 | 0 | 0 | 0 | 0 | 0 |
| FLCOM | #h1pdmrb | 0 | 0 | 1 | 0 | 1 | 0 | 0 | 0 | 0 | 1 | 0 | 1 | 0 | 0 | 0 | 0 | 0 | 0 |
| FLCOM | #h3 | 0 | 0 | 1 | 1 | 0 | 0 | 0 | 0 | 0 | 0 | 0 | 0 | 0 | 0 | 0 | 0 | 0 | 0 |
| FLCOM | #h3hmpvb | 0 | 0 | 1 | 1 | 0 | 0 | 0 | 0 | 1 | 0 | 0 | 0 | 0 | 0 | 0 | 0 | 0 | 0 |
| FLCOM | #h3ra | 0 | 0 | 1 | 1 | 0 | 0 | 0 | 0 | 0 | 1 | 1 | 0 | 0 | 0 | 0 | 0 | 0 | 0 |
| FLCOM | #h3rb | 0 | 0 | 1 | 1 | 0 | 0 | 0 | 0 | 0 | 1 | 0 | 1 | 0 | 0 | 0 | 0 | 0 | 0 |
| FLCOM | #hmpva | 0 | 0 | 0 | 0 | 0 | 0 | 0 | 0 | 1 | 0 | 0 | 0 | 0 | 0 | 0 | 0 | 0 | 0 |
| FLCOM | #hmpvab | 0 | 0 | 0 | 0 | 0 | 0 | 0 | 0 | 1 | 0 | 0 | 0 | 0 | 0 | 0 | 0 | 0 | 0 |
| FLCOM | #hmpvb | 0 | 0 | 0 | 0 | 0 | 0 | 0 | 0 | 1 | 0 | 0 | 0 | 0 | 0 | 0 | 0 | 0 | 0 |
| FLCOM | #laiv | 0 | 0 | 0 | 0 | 0 | 0 | 0 | 1 | 0 | 0 | 0 | 0 | 0 | 0 | 0 | 0 | 0 | 0 |
| FLCOM | #nvd | 0 | 1 | 0 | 0 | 0 | 0 | 0 | 0 | 0 | 0 | 0 | 0 | 0 | 0 | 0 | 0 | 0 | 0 |
| FLCOM | #ra | 0 | 0 | 0 | 0 | 0 | 0 | 0 | 0 | 0 | 1 | 1 | 0 | 0 | 0 | 0 | 0 | 0 | 0 |
| FLCOM | #rarb | 0 | 0 | 0 | 0 | 0 | 0 | 0 | 0 | 0 | 1 | 1 | 1 | 0 | 0 | 0 | 0 | 0 | 0 |
| FLCOM | #rb | 0 | 0 | 0 | 0 | 0 | 0 | 0 | 0 | 0 | 1 | 0 | 1 | 0 | 0 | 0 | 0 | 0 | 0 |
| FLCOM | #rbhmpvb | 0 | 0 | 0 | 0 | 0 | 0 | 0 | 0 | 1 | 1 | 0 | 1 | 0 | 0 | 0 | 0 | 0 | 0 |
| FLCOM | #un | 0 | 0 | 0 | 0 | 0 | 0 | 0 | 0 | 0 | 0 | 0 | 0 | 0 | 0 | 0 | 0 | 0 | 0 |
| FLCOM | #unad | 0 | 0 | 0 | 0 | 0 | 0 | 0 | 0 | 0 | 0 | 0 | 0 | 0 | 0 | 0 | 0 | 0 | 0 |
| FLCOM5_RCGP | #nt10 | 1 | 0 | 0 | 0 | 0 | 0 | 0 | 0 | 0 | 0 | 0 | 0 | 0 | 0 | 0 | 0 | 0 | 0 |
| FLCOM5_RCGP | #nt3 | 1 | 0 | 0 | 0 | 0 | 0 | 0 | 0 | 0 | 0 | 0 | 0 | 0 | 0 | 0 | 0 | 0 | 0 |
| FLCOM5_RCGP | #nt4 | 1 | 0 | 0 | 0 | 0 | 0 | 0 | 0 | 0 | 0 | 0 | 0 | 0 | 0 | 0 | 0 | 0 | 0 |
| FLCOM5_RCGP | #nt5 | 1 | 0 | 0 | 0 | 0 | 0 | 0 | 0 | 0 | 0 | 0 | 0 | 0 | 0 | 0 | 0 | 0 | 0 |
| FLCOM5_RCGP | #nt6 | 1 | 0 | 0 | 0 | 0 | 0 | 0 | 0 | 0 | 0 | 0 | 0 | 0 | 0 | 0 | 0 | 0 | 0 |
| FLCOM5_RCGP | #nt7 | 1 | 0 | 0 | 0 | 0 | 0 | 0 | 0 | 0 | 0 | 0 | 0 | 0 | 0 | 0 | 0 | 0 | 0 |
| FLCOM5_RCGP | #nt8 | 1 | 0 | 0 | 0 | 0 | 0 | 0 | 0 | 0 | 0 | 0 | 0 | 0 | 0 | 0 | 0 | 0 | 0 |
| FLCOM5_RCGP | #nt9 | 1 | 0 | 0 | 0 | 0 | 0 | 0 | 0 | 0 | 0 | 0 | 0 | 0 | 0 | 0 | 0 | 0 | 0 |
| FLCORC | COVID-19 confirmed | 0 | 0 | 0 | 0 | 0 | 0 | 0 | 0 | 0 | 0 | 0 | 0 | 0 | 1 | 0 | 0 | 0 | 0 |
| FLCORC | Indeterminate result for SARS CoV-2 PCR testing. Please send a second sample. | 0 | 0 | 0 | 0 | 0 | 0 | 0 | 0 | 0 | 0 | 0 | 0 | 0 | 0 | 0 | 0 | 0 | 0 |
| FLCORC | Not tested | 1 | 0 | 0 | 0 | 0 | 0 | 0 | 0 | 0 | 0 | 0 | 0 | 0 | 0 | 0 | 0 | 0 | 0 |
| FLCORC | SARS CoV- NOT detected in this sample | 0 | 1 | 0 | 0 | 0 | 0 | 0 | 0 | 0 | 0 | 0 | 0 | 0 | 0 | 0 | 0 | 0 | 0 |
| FLCORC | SARS CoV-2 detected (in this Nasopharyngeal Swab) | 0 | 0 | 0 | 0 | 0 | 0 | 0 | 0 | 0 | 0 | 0 | 0 | 0 | 1 | 0 | 0 | 0 | 0 |
| FLCORC | SARS CoV-2 detected in this sample | 0 | 0 | 0 | 0 | 0 | 0 | 0 | 0 | 0 | 0 | 0 | 0 | 0 | 1 | 0 | 0 | 0 | 0 |
| FLCORC | SARS CoV-2 detected in this samplecv6 | 0 | 0 | 0 | 0 | 0 | 0 | 0 | 0 | 0 | 0 | 0 | 0 | 0 | 1 | 0 | 0 | 0 | 0 |
| FLCORC | SARS CoV-2 detected in this sampleSample repeatedly weakly positive in a single PCR detector. Please interpret this result with caution due to the uncertainty of clinical significance | 0 | 0 | 0 | 0 | 0 | 0 | 0 | 0 | 0 | 0 | 0 | 0 | 0 | 0 | 0 | 0 | 0 | 0 |
| FLCORC | SARS CoV-2 NOT detected | 0 | 1 | 0 | 0 | 0 | 0 | 0 | 0 | 0 | 0 | 0 | 0 | 0 | 0 | 0 | 0 | 0 | 0 |
| FLCORC | SARS CoV-2 NOT detected in this sample | 0 | 1 | 0 | 0 | 0 | 0 | 0 | 0 | 0 | 0 | 0 | 0 | 0 | 0 | 0 | 0 | 0 | 0 |
| FLCORC | SARS CoV-2 NOT detected in this sample (dry swab) | 0 | 1 | 0 | 0 | 0 | 0 | 0 | 0 | 0 | 0 | 0 | 0 | 0 | 0 | 0 | 0 | 0 | 0 |
| FLCORC | SARS CoV-2 NOT detectedin this sample | 0 | 1 | 0 | 0 | 0 | 0 | 0 | 0 | 0 | 0 | 0 | 0 | 0 | 0 | 0 | 0 | 0 | 0 |
| FLCORC | Unable to confirm the presence of SARS CoV-2 in samples submitted. | 0 | 0 | 0 | 0 | 0 | 0 | 0 | 0 | 0 | 0 | 0 | 0 | 0 | 0 | 0 | 0 | 0 | 0 |
| FLHRV | #d | 0 | 0 | 0 | 0 | 0 | 0 | 0 | 0 | 0 | 0 | 0 | 0 | 0 | 0 | 0 | 0 | 1 | 0 |
| FLHRV | #inv | 0 | 0 | 0 | 0 | 0 | 0 | 0 | 0 | 0 | 0 | 0 | 0 | 0 | 0 | 0 | 0 | 0 | 0 |
| FLHRV | #nd | 0 | 1 | 0 | 0 | 0 | 0 | 0 | 0 | 0 | 0 | 0 | 0 | 0 | 0 | 0 | 0 | 0 | 0 |
| FLHRV | #nt | 1 | 0 | 0 | 0 | 0 | 0 | 0 | 0 | 0 | 0 | 0 | 0 | 0 | 0 | 0 | 0 | 0 | 0 |
| FLU_COMMENT | #h3 | 0 | 0 | 1 | 1 | 0 | 0 | 0 | 0 | 0 | 0 | 0 | 0 | 0 | 0 | 0 | 0 | 0 | 0 |
| FLU_COMMENT | #hmpv | 0 | 0 | 0 | 0 | 0 | 0 | 0 | 0 | 1 | 0 | 0 | 0 | 0 | 0 | 0 | 0 | 0 | 0 |
| FLU_COMMENT | #nt | 1 | 0 | 0 | 0 | 0 | 0 | 0 | 0 | 0 | 0 | 0 | 0 | 0 | 0 | 0 | 0 | 0 | 0 |
| FLU_COMMENT | #nvd | 0 | 1 | 0 | 0 | 0 | 0 | 0 | 0 | 0 | 0 | 0 | 0 | 0 | 0 | 0 | 0 | 0 | 0 |
| FLU_COMMENT | #ra | 0 | 0 | 0 | 0 | 0 | 0 | 0 | 0 | 0 | 1 | 1 | 0 | 0 | 0 | 0 | 0 | 0 | 0 |
| FLU_COMMENT | #rahmpv | 0 | 0 | 0 | 0 | 0 | 0 | 0 | 0 | 1 | 1 | 1 | 0 | 0 | 0 | 0 | 0 | 0 | 0 |
| FLU_COMMENT | #rb | 0 | 0 | 0 | 0 | 0 | 0 | 0 | 0 | 0 | 1 | 0 | 1 | 0 | 0 | 0 | 0 | 0 | 0 |
| FLU_COMMENT | Both RSVB and Adenovirus were detected in this sample. | 0 | 0 | 0 | 0 | 0 | 0 | 0 | 0 | 0 | 1 | 0 | 1 | 0 | 0 | 0 | 1 | 0 | 0 |
| FLU_COMMENT | hMPV | 0 | 0 | 0 | 0 | 0 | 0 | 0 | 0 | 1 | 0 | 0 | 0 | 0 | 0 | 0 | 0 | 0 | 0 |
| FLU_COMMENT | hMPV and human seasonal coronavirus OC43 detected | 0 | 0 | 0 | 0 | 0 | 0 | 0 | 0 | 1 | 0 | 0 | 0 | 0 | 0 | 1 | 0 | 0 | 0 |
| FLU_COMMENT | hMPV detected | 0 | 0 | 0 | 0 | 0 | 0 | 0 | 0 | 1 | 0 | 0 | 0 | 0 | 0 | 0 | 0 | 0 | 0 |
| FLU_COMMENT | HMPV detected, Human coronavirus OC43 detected, Influenza A detected. | 0 | 0 | 1 | 0 | 0 | 1 | 0 | 0 | 1 | 0 | 0 | 0 | 0 | 0 | 1 | 0 | 0 | 0 |
| FLU_COMMENT | Human seasonal coronavirus #NL63 deteceted | 0 | 0 | 0 | 0 | 0 | 0 | 0 | 0 | 0 | 0 | 0 | 0 | 0 | 0 | 1 | 0 | 0 | 0 |
| FLU_COMMENT | Human seasonal coronavirus 229E detected. | 0 | 0 | 0 | 0 | 0 | 0 | 0 | 0 | 0 | 0 | 0 | 0 | 0 | 0 | 1 | 0 | 0 | 0 |
| FLU_COMMENT | Human seasonal coronavirus HKU1 and OC43 detected. | 0 | 0 | 0 | 0 | 0 | 0 | 0 | 0 | 0 | 0 | 0 | 0 | 0 | 0 | 1 | 0 | 0 | 0 |
| FLU_COMMENT | Human seasonal coronavirus HKU1 detected in this sample | 0 | 0 | 0 | 0 | 0 | 0 | 0 | 0 | 0 | 0 | 0 | 0 | 0 | 0 | 1 | 0 | 0 | 0 |
| FLU_COMMENT | Human seasonal coronavirus HKU1 detected. | 0 | 0 | 0 | 0 | 0 | 0 | 0 | 0 | 0 | 0 | 0 | 0 | 0 | 0 | 1 | 0 | 0 | 0 |
| FLU_COMMENT | Human seasonal coronavirus NL63 and hMPV detected. | 0 | 0 | 0 | 0 | 0 | 0 | 0 | 0 | 1 | 0 | 0 | 0 | 0 | 0 | 1 | 0 | 0 | 0 |
| FLU_COMMENT | Human seasonal coronavirus NL63 and OC43 detected. | 0 | 0 | 0 | 0 | 0 | 0 | 0 | 0 | 0 | 0 | 0 | 0 | 0 | 0 | 1 | 0 | 0 | 0 |
| FLU_COMMENT | Human seasonal coronavirus NL63 and RSVB detected. | 0 | 0 | 0 | 0 | 0 | 0 | 0 | 0 | 0 | 1 | 0 | 1 | 0 | 0 | 1 | 0 | 0 | 0 |
| FLU_COMMENT | Human seasonal coronavirus NL63 detected in this sample | 0 | 0 | 0 | 0 | 0 | 0 | 0 | 0 | 0 | 0 | 0 | 0 | 0 | 0 | 1 | 0 | 0 | 0 |
| FLU_COMMENT | Human seasonal coronavirus NL63 detected. | 0 | 0 | 0 | 0 | 0 | 0 | 0 | 0 | 0 | 0 | 0 | 0 | 0 | 0 | 1 | 0 | 0 | 0 |
| FLU_COMMENT | Human seasonal coronavirus OC43 and hMPV detected. | 0 | 0 | 0 | 0 | 0 | 0 | 0 | 0 | 1 | 0 | 0 | 0 | 0 | 0 | 1 | 0 | 0 | 0 |
| FLU_COMMENT | Human seasonal coronavirus OC43 and Influenza A H3 detected. | 0 | 0 | 1 | 1 | 0 | 0 | 0 | 0 | 0 | 0 | 0 | 0 | 0 | 0 | 1 | 0 | 0 | 0 |
| FLU_COMMENT | Human seasonal coronavirus OC43 and Influenza A subtype (H1)pdm09 detected. | 0 | 0 | 1 | 0 | 1 | 0 | 0 | 0 | 0 | 0 | 0 | 0 | 0 | 0 | 1 | 0 | 0 | 0 |
| FLU_COMMENT | Human seasonal coronavirus OC43 and Influenza A subtype H3 detected. | 0 | 0 | 1 | 1 | 0 | 0 | 0 | 0 | 0 | 0 | 0 | 0 | 0 | 0 | 1 | 0 | 0 | 0 |
| FLU_COMMENT | Human seasonal coronavirus OC43 and Influenza B virus detected. | 0 | 0 | 0 | 0 | 0 | 0 | 1 | 0 | 0 | 0 | 0 | 0 | 0 | 0 | 1 | 0 | 0 | 0 |
| FLU_COMMENT | Human seasonal coronavirus OC43 and RSVA detected | 0 | 0 | 0 | 0 | 0 | 0 | 0 | 0 | 0 | 1 | 1 | 0 | 0 | 0 | 1 | 0 | 0 | 0 |
| FLU_COMMENT | Human seasonal coronavirus OC43 and RSVA detected. | 0 | 0 | 0 | 0 | 0 | 0 | 0 | 0 | 0 | 1 | 1 | 0 | 0 | 0 | 1 | 0 | 0 | 0 |
| FLU_COMMENT | Human seasonal coronavirus OC43 and RSVB detected. | 0 | 0 | 0 | 0 | 0 | 0 | 0 | 0 | 0 | 1 | 0 | 1 | 0 | 0 | 1 | 0 | 0 | 0 |
| FLU_COMMENT | Human seasonal coronavirus OC43 detected. | 0 | 0 | 0 | 0 | 0 | 0 | 0 | 0 | 0 | 0 | 0 | 0 | 0 | 0 | 1 | 0 | 0 | 0 |
| FLU_COMMENT | Human seasonal coronavirus OC43 detected. Influenza A subtype H1 detected in this sample. | 0 | 0 | 1 | 0 | 1 | 0 | 0 | 0 | 0 | 0 | 0 | 0 | 0 | 0 | 1 | 0 | 0 | 0 |
| FLU_COMMENT | Human seasonal coronaviruses 229E and NL63 detected. | 0 | 0 | 0 | 0 | 0 | 0 | 0 | 0 | 0 | 0 | 0 | 0 | 0 | 0 | 1 | 0 | 0 | 0 |
| FLU_COMMENT | Human seasonal coronaviruses NL63 and OC43 detected. RSV B detected in this sample. | 0 | 0 | 0 | 0 | 0 | 0 | 0 | 0 | 0 | 1 | 0 | 1 | 0 | 0 | 1 | 0 | 0 | 0 |
| FLU_COMMENT | Influenza A and RSVA detected | 0 | 0 | 1 | 0 | 0 | 1 | 0 | 0 | 0 | 1 | 1 | 0 | 0 | 0 | 0 | 0 | 0 | 0 |
| FLU_COMMENT | Influenza A and RSVB detected | 0 | 0 | 1 | 0 | 0 | 1 | 0 | 0 | 0 | 1 | 0 | 1 | 0 | 0 | 0 | 0 | 0 | 0 |
| FLU_COMMENT | Influenza A and subtype (H1)pdm09, H3 and Influenza B detected | 0 | 0 | 1 | 1 | 1 | 0 | 1 | 0 | 0 | 0 | 0 | 0 | 0 | 0 | 0 | 0 | 0 | 0 |
| FLU_COMMENT | Influenza A subtype (H1)pdm09 and H3 detected | 0 | 0 | 1 | 1 | 1 | 0 | 0 | 0 | 0 | 0 | 0 | 0 | 0 | 0 | 0 | 0 | 0 | 0 |
| FLU_COMMENT | Influenza A subtype (H1)pdm09 and hMPV detected | 0 | 0 | 1 | 0 | 1 | 0 | 0 | 0 | 1 | 0 | 0 | 0 | 0 | 0 | 0 | 0 | 0 | 0 |
| FLU_COMMENT | Influenza A subtype (H1)pdm09 and RSVA detected | 0 | 0 | 1 | 0 | 1 | 0 | 0 | 0 | 0 | 1 | 1 | 0 | 0 | 0 | 0 | 0 | 0 | 0 |
| FLU_COMMENT | Influenza A subtype (H1)pdm09 and RSVB detected | 0 | 0 | 1 | 0 | 1 | 0 | 0 | 0 | 0 | 1 | 0 | 1 | 0 | 0 | 0 | 0 | 0 | 0 |
| FLU_COMMENT | Influenza A subtype (H1)pdm09 detected | 0 | 0 | 1 | 0 | 1 | 0 | 0 | 0 | 0 | 0 | 0 | 0 | 0 | 0 | 0 | 0 | 0 | 0 |
| FLU_COMMENT | Influenza A subtype (H1)pdm09 detected and human coronavirus NL63 detected. | 0 | 0 | 1 | 0 | 1 | 0 | 0 | 0 | 0 | 0 | 0 | 0 | 0 | 0 | 1 | 0 | 0 | 0 |
| FLU_COMMENT | Influenza A subtype H3 and hMPV detected | 0 | 0 | 1 | 1 | 0 | 0 | 0 | 0 | 1 | 0 | 0 | 0 | 0 | 0 | 0 | 0 | 0 | 0 |
| FLU_COMMENT | Influenza A subtype H3 and Human Coronavirus NL63 detected | 0 | 0 | 1 | 1 | 0 | 0 | 0 | 0 | 0 | 0 | 0 | 0 | 0 | 0 | 1 | 0 | 0 | 0 |
| FLU_COMMENT | Influenza A subtype H3 and human seasonal coronavirus NL63 detected | 0 | 0 | 1 | 1 | 0 | 0 | 0 | 0 | 0 | 0 | 0 | 0 | 0 | 0 | 1 | 0 | 0 | 0 |
| FLU_COMMENT | Influenza A subtype H3 and human seasonal coronavirus OC43 detected | 0 | 0 | 1 | 1 | 0 | 0 | 0 | 0 | 0 | 0 | 0 | 0 | 0 | 0 | 1 | 0 | 0 | 0 |
| FLU_COMMENT | Influenza A subtype H3 and Influenza B detected | 0 | 0 | 1 | 1 | 0 | 0 | 1 | 0 | 0 | 0 | 0 | 0 | 0 | 0 | 0 | 0 | 0 | 0 |
| FLU_COMMENT | Influenza A subtype H3 and RSVA detected | 0 | 0 | 1 | 1 | 0 | 0 | 0 | 0 | 0 | 1 | 1 | 0 | 0 | 0 | 0 | 0 | 0 | 0 |
| FLU_COMMENT | Influenza A subtype H3 and RSVB detected | 0 | 0 | 1 | 1 | 0 | 0 | 0 | 0 | 0 | 1 | 0 | 1 | 0 | 0 | 0 | 0 | 0 | 0 |
| FLU_COMMENT | Influenza A subtype H3 detected | 0 | 0 | 1 | 1 | 0 | 0 | 0 | 0 | 0 | 0 | 0 | 0 | 0 | 0 | 0 | 0 | 0 | 0 |
| FLU_COMMENT | Influenza A subtype H3 detected and human coronavirus OC43 detected. | 0 | 0 | 1 | 1 | 0 | 0 | 0 | 0 | 0 | 0 | 0 | 0 | 0 | 0 | 1 | 0 | 0 | 0 |
| FLU_COMMENT | Influenza A subtype H3 detected. Human seasonal coronavirus NL63 detected. | 0 | 0 | 1 | 1 | 0 | 0 | 0 | 0 | 0 | 0 | 0 | 0 | 0 | 0 | 1 | 0 | 0 | 0 |
| FLU_COMMENT | Influenza A subtype H3 detected. Human seasonal coronavirus OC43 detected in this sample. | 0 | 0 | 1 | 1 | 0 | 0 | 0 | 0 | 0 | 0 | 0 | 0 | 0 | 0 | 1 | 0 | 0 | 0 |
| FLU_COMMENT | Influenza A subtype H3 detected. Human seasonal coronavirus OC43 detected. | 0 | 0 | 1 | 1 | 0 | 0 | 0 | 0 | 0 | 0 | 0 | 0 | 0 | 0 | 1 | 0 | 0 | 0 |
| FLU_COMMENT | Influenza A subtype H3, RSVA and hMPV detected | 0 | 0 | 1 | 1 | 0 | 0 | 0 | 0 | 1 | 1 | 1 | 0 | 0 | 0 | 0 | 0 | 0 | 0 |
| FLU_COMMENT | Influenza A virus and RSVB detected | 0 | 0 | 1 | 0 | 0 | 1 | 0 | 0 | 0 | 1 | 0 | 1 | 0 | 0 | 0 | 0 | 0 | 0 |
| FLU_COMMENT | Influenza A virus detected | 0 | 0 | 1 | 0 | 0 | 1 | 0 | 0 | 0 | 0 | 0 | 0 | 0 | 0 | 0 | 0 | 0 | 0 |
| FLU_COMMENT | Influenza B and hMPV detected | 0 | 0 | 0 | 0 | 0 | 0 | 1 | 0 | 1 | 0 | 0 | 0 | 0 | 0 | 0 | 0 | 0 | 0 |
| FLU_COMMENT | Influenza B and RSVA detected | 0 | 0 | 0 | 0 | 0 | 0 | 1 | 0 | 0 | 1 | 1 | 0 | 0 | 0 | 0 | 0 | 0 | 0 |
| FLU_COMMENT | Influenza B and RSVB detected | 0 | 0 | 0 | 0 | 0 | 0 | 1 | 0 | 0 | 1 | 0 | 1 | 0 | 0 | 0 | 0 | 0 | 0 |
| FLU_COMMENT | Influenza B virus and human seasonal coronavirus OC43 detected | 0 | 0 | 0 | 0 | 0 | 0 | 1 | 0 | 0 | 0 | 0 | 0 | 0 | 0 | 1 | 0 | 0 | 0 |
| FLU_COMMENT | Influenza B virus detected | 0 | 0 | 0 | 0 | 0 | 0 | 1 | 0 | 0 | 0 | 0 | 0 | 0 | 0 | 0 | 0 | 0 | 0 |
| FLU_COMMENT | Live attenuated influenza vaccine strains detected by PCR | 0 | 0 | 0 | 0 | 0 | 0 | 0 | 1 | 0 | 0 | 0 | 0 | 0 | 0 | 0 | 0 | 0 | 0 |
| FLU_COMMENT | No detection of seasonal respiratory virus (seasonal influenza, hMPV, RSV, seasonal human coronavirus) | 0 | 1 | 0 | 0 | 0 | 0 | 0 | 0 | 0 | 0 | 0 | 0 | 0 | 0 | 0 | 0 | 0 | 0 |
| FLU_COMMENT | No seasonal respiratory virus detected | 0 | 1 | 0 | 0 | 0 | 0 | 0 | 0 | 0 | 0 | 0 | 0 | 0 | 0 | 0 | 0 | 0 | 0 |
| FLU_COMMENT | No virus detected | 0 | 1 | 0 | 0 | 0 | 0 | 0 | 0 | 0 | 0 | 0 | 0 | 0 | 0 | 0 | 0 | 0 | 0 |
| FLU_COMMENT | Not tested | 1 | 0 | 0 | 0 | 0 | 0 | 0 | 0 | 0 | 0 | 0 | 0 | 0 | 0 | 0 | 0 | 0 | 0 |
| FLU_COMMENT | RSV A | 0 | 0 | 0 | 0 | 0 | 0 | 0 | 0 | 0 | 1 | 1 | 0 | 0 | 0 | 0 | 0 | 0 | 0 |
| FLU_COMMENT | RSV B | 0 | 0 | 0 | 0 | 0 | 0 | 0 | 0 | 0 | 1 | 0 | 1 | 0 | 0 | 0 | 0 | 0 | 0 |
| FLU_COMMENT | RSVA and hMPV detected | 0 | 0 | 0 | 0 | 0 | 0 | 0 | 0 | 1 | 1 | 1 | 0 | 0 | 0 | 0 | 0 | 0 | 0 |
| FLU_COMMENT | RSVA and human seasonal coronavirus OC43 detected | 0 | 0 | 0 | 0 | 0 | 0 | 0 | 0 | 0 | 1 | 1 | 0 | 0 | 0 | 1 | 0 | 0 | 0 |
| FLU_COMMENT | RSVA and RSVB detected | 0 | 0 | 0 | 0 | 0 | 0 | 0 | 0 | 0 | 1 | 1 | 1 | 0 | 0 | 0 | 0 | 0 | 0 |
| FLU_COMMENT | RSVA detected | 0 | 0 | 0 | 0 | 0 | 0 | 0 | 0 | 0 | 1 | 1 | 0 | 0 | 0 | 0 | 0 | 0 | 0 |
| FLU_COMMENT | RSVB and hMPV detected | 0 | 0 | 0 | 0 | 0 | 0 | 0 | 0 | 1 | 1 | 0 | 1 | 0 | 0 | 0 | 0 | 0 | 0 |
| FLU_COMMENT | RSVB detected | 0 | 0 | 0 | 0 | 0 | 0 | 0 | 0 | 0 | 1 | 0 | 1 | 0 | 0 | 0 | 0 | 0 | 0 |
| FLU_COMMENT | RSVB detected in this sample. Human seasonal coronavirus OC43 detected. | 0 | 0 | 0 | 0 | 0 | 0 | 0 | 0 | 0 | 1 | 0 | 1 | 0 | 0 | 1 | 0 | 0 | 0 |
| FLU_COMMENT | RSVB detected. Human seasonal coronavirus 229E detected. | 0 | 0 | 0 | 0 | 0 | 0 | 0 | 0 | 0 | 1 | 0 | 1 | 0 | 0 | 1 | 0 | 0 | 0 |
| FLU_COMMENT | RSVB, human seasonal coronavirus NL63 and influenza A subtype (H1)pdm09 detected. | 0 | 0 | 1 | 0 | 1 | 0 | 0 | 0 | 0 | 0 | 0 | 0 | 0 | 0 | 1 | 0 | 0 | 0 |
| FLU_COMMENT | Sample unsuitable for testing | 1 | 0 | 0 | 0 | 0 | 0 | 0 | 0 | 0 | 0 | 0 | 0 | 0 | 0 | 0 | 0 | 0 | 0 |
| FLU_COMMENT | Seasonal coronavirus HKU1 and human metapneumovirus detected | 0 | 0 | 0 | 0 | 0 | 0 | 0 | 0 | 1 | 0 | 0 | 0 | 0 | 0 | 1 | 0 | 0 | 0 |
| FLU_COMMENT | Suspected detection of live attenuated influenza vaccine strain/s, but unable to confirm by whole genome sequencing. | 0 | 0 | 0 | 0 | 0 | 0 | 0 | 0 | 0 | 0 | 0 | 0 | 0 | 0 | 0 | 0 | 0 | 0 |
| FLU_COMMENT | This sample showed equivocal result for Human seasonal coronavirus NL63.Please interpret results with caution due to uncertainty of clinical significance | 0 | 0 | 0 | 0 | 0 | 0 | 0 | 0 | 0 | 0 | 0 | 0 | 0 | 0 | 1 | 0 | 0 | 0 |
| FLU_COMMENT | Unable to confirm result(s). Please send an additional sample. | 0 | 0 | 0 | 0 | 0 | 0 | 0 | 0 | 0 | 0 | 0 | 0 | 0 | 0 | 0 | 0 | 0 | 0 |
| SeasonalCoronavirusResult | #229E | 0 | 0 | 0 | 0 | 0 | 0 | 0 | 0 | 0 | 0 | 0 | 0 | 0 | 0 | 1 | 0 | 0 | 0 |
| SeasonalCoronavirusResult | #229E#HKU1 | 0 | 0 | 0 | 0 | 0 | 0 | 0 | 0 | 0 | 0 | 0 | 0 | 0 | 0 | 1 | 0 | 0 | 0 |
| SeasonalCoronavirusResult | #229E#NL63 | 0 | 0 | 0 | 0 | 0 | 0 | 0 | 0 | 0 | 0 | 0 | 0 | 0 | 0 | 1 | 0 | 0 | 0 |
| SeasonalCoronavirusResult | #HKU1 | 0 | 0 | 0 | 0 | 0 | 0 | 0 | 0 | 0 | 0 | 0 | 0 | 0 | 0 | 1 | 0 | 0 | 0 |
| SeasonalCoronavirusResult | #HKU1#OC43 | 0 | 0 | 0 | 0 | 0 | 0 | 0 | 0 | 0 | 0 | 0 | 0 | 0 | 0 | 1 | 0 | 0 | 0 |
| SeasonalCoronavirusResult | #NL63 | 0 | 0 | 0 | 0 | 0 | 0 | 0 | 0 | 0 | 0 | 0 | 0 | 0 | 0 | 1 | 0 | 0 | 0 |
| SeasonalCoronavirusResult | #NL63#OC43 | 0 | 0 | 0 | 0 | 0 | 0 | 0 | 0 | 0 | 0 | 0 | 0 | 0 | 0 | 1 | 0 | 0 | 0 |
| SeasonalCoronavirusResult | #nt | 1 | 0 | 0 | 0 | 0 | 0 | 0 | 0 | 0 | 0 | 0 | 0 | 0 | 0 | 0 | 0 | 0 | 0 |
| SeasonalCoronavirusResult | #nvd | 0 | 1 | 0 | 0 | 0 | 0 | 0 | 0 | 0 | 0 | 0 | 0 | 0 | 0 | 0 | 0 | 0 | 0 |
| SeasonalCoronavirusResult | #OC43 | 0 | 0 | 0 | 0 | 0 | 0 | 0 | 0 | 0 | 0 | 0 | 0 | 0 | 0 | 1 | 0 | 0 | 0 |
| SeasonalCoronavirusResult | ADDED | 0 | 0 | 0 | 0 | 0 | 0 | 0 | 0 | 0 | 0 | 0 | 0 | 0 | 0 | 0 | 0 | 0 | 0 |

The table maps the free-text virology results in the “ColumnValue” column to the cleaned binary output columns, from “NotTested” onwards (left to right).
